# 4449 Building Capacity in the Flint Community in the Midst of the Ongoing Water Crisis

**DOI:** 10.1017/cts.2020.262

**Published:** 2020-07-29

**Authors:** Athena S. McKay, Adam Paberzs, Patricia Piechowski, Donald Vereen, Susan Woolford

**Affiliations:** 1 University of Michigan; 2 MICHR Community Engagement

## Abstract

OBJECTIVES/GOALS: Examining the impact of the Building Capacity for Research and Action (BCRA) Award created by the Community Engagement (CE) Program at the Michigan Institute for Clinical & Health Research (MICHR)--a Clinical & Translational Science Award (CTSA) site at the University of Michigan--in partnership with Community Based Organization Partners (CBOP). METHODS/STUDY POPULATION: The BCRA is a funding mechanism that supports new community-engaged research (CEnR) partnerships and projects that address community-identified health needs in Flint, Michigan. BCRA projects are required to be Flint-based and inclusive of both community and academic partners. A study section consisting of 10 MICHR-affiliated faculty and community partners reviewed proposals and made funding decisions. Funded teams were trained on Institutional Review Board (IRB) and reporting requirements by CE staff. MICHR provides support to BCRA-funded teams through monthly email correspondence with the CE Flint connector, budget review, mediation, regulatory assurance of IRB and the National Center for Advancing Translational Science (NCATS) requirements, coordinating six-month and final reporting, and hosting an annual stakeholder meet and greet. RESULTS/ANTICIPATED RESULTS: In 2017, the BCRA Award submitted its first request for proposals. It received 20 applications in 2018, and selected eight awardees, providing them with a total of $60,000 in funding. Four received $5,000 for partnership development and another four received $10,000 for their research projects. The BCRA Award received 16 applications in 2019, expanding its academic pool to include the University of Chicago, U-M Flint, Michigan State University, and Michigan State University-Flint in addition to the University of Michigan. Five recipients were selected and received a total of $45,000 in funding. One was awarded $5,000 for partnership development and another four were awarded $10,000 for their research projects. MICHR has invested over $100,000 in Flint through this mechanism, which was renewed in 2019. DISCUSSION/SIGNIFICANCE OF IMPACT: Each awardee presented at the annual stakeholder meet and greet. They showcased their projects with a brief overview and spoke about their expectations, lessons learned, partnership strengths and challenges, translational issues, and proposed next steps for subsequent grants, publications.

